# New Method for Determining Mode-I Static Fracture Toughness of Coal Using Particles

**DOI:** 10.3390/ma17081765

**Published:** 2024-04-11

**Authors:** Qiang Cheng, Gun Huang, Jie Zheng, Qinming Liang

**Affiliations:** 1 State Key Laboratory of Coal Mine Disaster Dynamics and Control, Chongqing University, Chongqing 400030, China; qiangcheng@cqu.edu.cn (Q.C.); qmliang@cqu.edu.cn (Q.L.); 2 School of Resources and Safety Engineering, Chongqing University, Chongqing 400030, China; 3 School of Geology and Mining Engineering, Xinjiang University, Urumqi 830046, China; jieuan@163.com

**Keywords:** coal, mechanical properties, fracture toughness, hardness coefficient, newly added surface area

## Abstract

Understanding the mechanical properties of coal is crucial for efficient mining and disaster prevention in coal mines. Coal contains numerous cracks and fissures, resulting in low strength and challenges in preparing standard samples for testing coal fracture toughness. In engineering, indicators such as the hardness coefficient (f value) and Hardgrove grindability index (HGI) are straightforward to measure. Various experiments, including drop weight, grinding, uniaxial compressive strength and three-point bending experiments, were conducted using notched semi-circular bend (NSCB) specimens and particle sizes of 1–2 mm/0.425–1 mm. Theoretical and experimental results indicate that the hardness coefficient of coal and rock is proportional to the crushing work ratio and inversely proportional to the mean equivalent diameter. Moreover, the square of the fracture toughness of coal and rock is directly proportional to the crushing work ratio, inversely proportional to the newly added area, directly proportional to the mean equivalent diameter and directly proportional to the hardness coefficient. The Mode-I fracture toughness of coal and rock can be rapidly determined through the density, the equivalent diameter after crushing and the elastic modulus, with experimental verification of its accuracy. Considering that smaller particle sizes exhibit greater resistance to breakage, the distribution mode of new surface areas after particle breakage was established, influenced by the initial particle size and the energy of a single broken particle. This study can assist in quickly and accurately determining the fracture toughness of coal.

## 1. Introduction

In recent years, the rapid progress in deep geological engineering—including shale gas and coalbed methane exploration, deep mineral extraction, nuclear waste burial, geothermal resource development and underground carbon dioxide storage—has garnered increasing attention toward the characteristics of rock and coal. Generally, coal plays a pivotal role as the primary energy source in the world, and it is extensively utilized in power generation, metallurgy, the chemical industry and various other essential sectors. As coal resources are being exploited at greater depths, the incidence of coal mine gas dynamic disasters, such as coal gas outbursts, has intensified [1]. The mechanical properties of coal mass significantly influence the occurrence and progression of coal mine gas dynamic disasters [2]. Elasticity parameters describe the elastic deformation of rock under load, while strength represents the critical stress at which rock failure occurs. Fracture toughness, closely associated with strength, measures the rock’s resistance to crack propagation and is crucial in analyzing brittle fracture growth [3]. Rock breakage is best described by tensile-based rock mechanics tests, and particularly Mode-I fracture toughness [4]. In coal mine safety, the hardness coefficient (f value) denotes coal breakability; the Hardgrove grindability index (HGI) signifies grindability; and hardness-coefficient-related indicators aid in preventing and controlling coal gas outbursts. The hardness coefficient and HGI are straightforward to measure. For easy prediction of coal fracture behavior, Bhagat demonstrated a significant correlation between fracture toughness and 1/HGI [5]. Consequently, a comprehensive understanding of coal fracture behavior is essential for coal mine gas dynamic disasters. Exploring the relationship between the f value and fracture toughness holds important implications for preventing and controlling coal mine gas dynamic disasters.

Ken P. Chong’s study on fracture toughness determination of layered materials concluded that linear elastic fracture mechanics is valid for anisotropic rock materials [6]. Numerous methods for determining Mode-I fracture toughness exist in the literature, with reviews on their attributes, advantages and drawbacks provided by Whittaker et al. [7] and Bearman [4]. To obtain precise, accurate and consistent results, the International Society for Rock Mechanics (ISRM) recommends four test procedures: (1) chevron bend (CB); (2) short rod (SR); (3) cracked chevron notched Brazilian disc (CCNBD); and (4) notched semi-circular bend (SCB) [8,9,10,11,12,13]. These standards outline sample preparation, dimensions and test procedures, including loading type and rate. They also provide formulae for fracture toughness from failure load and geometrical factors [3]. Mohammad Reza Mohammad Aliha et al. examined the effect of specimen type on tensile fracture toughness of rock materials, finding significant dependence of Mode-I fracture toughness on specimen geometry and loading type [14]. Morteza Nejati et al. modified the semi-circular bend test to determine the fracture toughness of anisotropic rocks and illustrated Mode-I fracture growth in anisotropic rocks [15,16,17].

For dynamic static fracture characteristics, Yin et al. investigated the fracture mechanism of coal rock using three-point bending tests under different gas pressures. They found that Mode-I fracture toughness and rupture energy decrease in coal is influenced by gas [18]. The use of more than one type of specimen is regarded as appropriate when it is required to measure the fracture toughness of anisotropic materials in different material directions of a rock sample [8]. Wang et al. examined the influence of bedding planes on both Mode-I and mixed-mode (I–II) dynamic fracture toughness of coal [19]. For coal, the fracture toughness measured at different scales and different orientations varies significantly. The fracture toughness of anisotropic materials varies elliptically with the bedding angle [20]. Shi et al. considered the co-effects of bedding planes and loading conditions on Mode-I fracture toughness of anisotropic rocks. The experimental results indicated that the bedding effects are the most obvious under static loading and become weaker as the loading rates increase [1]. Sun et al. investigated the combined impact of specimen size and anisotropy on the Mode-I fracture toughness of coal and developed a size effect model for fracture toughness considering micro-cracks and bedding angle, as well as an anisotropy model accounting for specimen size [21]. For dynamic fracture characteristics of coal, Wang et al. studied the dynamic toughness of coal with a bedding structure based on the NSCB impact test [22].

The macro-mechanical properties of coal are closely related to its microscopic mechanical properties. Nanoindentation experiments, as utilized by Liu et al., offer an efficient and precise approach for investigating the micro-mechanical properties of soft and fractured coal at the nanoscale [23]. Moreover, Ma et al. highlighted the growing popularity of nanoindentation as a method to determine mechanical properties in both homogeneous and heterogeneous materials [24]. G.L. Manjunath et al. introduced a model to estimate the micro-scale fracture toughness of Gondwana coal using nanoindentation, calculating the fracture energy and fracture area from pop-in events in the loading curve [25]. Sun et al. investigated the mechanical properties of crushed coal samples based on the nanoindentation technique [26]. Liu Peng et al. utilized nanoindentation tests to probe the nano-mechanical behavior of coal [27]. Meng et al. investigated the mechanical properties and failure mechanisms of different rank coals at the nanoscale [28]. These results revealed clear linear relationships between nanoindentation hardness, the elastic modulus and fracture toughness of coal [26,27,28].

Fracture toughness denotes the critical stress intensity factor threshold beyond which catastrophic crack propagation occurs. In rock comminution, the intrinsic tensile property, measured as fracture toughness, often governs the breakage of individual rock particles. Breakage in comminution is itself mainly due to compression-induced tensile failure [4]. In linear elastic fracture mechanics, fracture toughness is closely linked to Griffith’s concept of fracture energy. According to Griffith’s theory, the strain energy released during fracture growth creates fracture surfaces. The surface area of particles during coal particle crushing is accessible to calculate when testing the f value and HGI. Cai et al. experimentally analyzed the relationship between Mode-I static fracture toughness and newly added surface area after crushing [29].

Particle size distributions and energy conversion during fragmentation are two of the principal problems in coal particle fragmentation research. Hossein Bayat reviewed particle size distribution models, along with their characteristics and fitting capabilities, mainly including power law models, exponential power models, logarithmic models, hyperbolic models, statistical distribution models, logarithmic exponential models and fractal models [30,31]. The distribution models after particle crushing currently include the Gates–Gaudin–Schuhmann (GGS) model, Rosin–Rammler (RR) model, lognormal model, normal model, etc. [32,33]. Jiang et al. investigated the effects of impact velocity on the energy and size distribution of rock crushing [34]. Luo et al. examined the relationship between the distribution of micro-coal particles and the crushing energy [35]. Wang et al. conducted impact crushing experiments and proposed a new fractal-theory-based index for evaluating rock firmness, calculating the surface areas of coal particles after impact crushing using the fractal particle size distribution theory [36]. Li et al. investigated the drop weight impact fragmentation of gas-containing coal particles. They supported the fractal particle size distribution model, which can most effectively describe the crushed coal particle sizes [37]. The above particle size distribution models mostly describe the volume distribution of particles after crushing. However, when using the newly added surface area after particle crushing to estimate fracture toughness, there is a lack of analysis of the distribution of the newly added surface area with different particle sizes after coal rock crushing.

This article conducted experiments using marble, sandstone, non-outburst raw coal and outburst raw coal. Various tests, including drop weight, grinding, uniaxial compressive strength and three-point bending experiments, were performed. Theoretical derivations established the relationship between fracture toughness, hardness coefficient (f value) and HGI. A novel method for measuring fracture toughness was developed, and the correlation between fracture toughness and new surface area was deduced. Additionally, the distribution pattern of new surface area after particle crushing was analyzed. The distribution of broken coal aids in the application of the compacting of coals and crushing of coals [38,39]. Simultaneously, the relationship between the f value and fracture toughness contributes to the prevention and mitigation of dynamic-gas-related disasters in coal mines.

## 2. Relationship between Fracture Toughness and Hardness Coefficient

Linear elastic fracture mechanics posits that when the energy dissipation during crack expansion is disregarded, and the crack does not bifurcate during expansion, the strain energy release rate of a Type I crack under a plane stress state is given by [13,40]

(1) $$G=\frac{K_{I}^{2}}{E}$$

where G is the strain energy release rate (J/mm^2^); $K_{I}$ is the fracture toughness (MPa·m^1/2^); E is the elastic modulus (GPa).

When the strain energy release rate is G=2γ, the crack propagates stably. Equation (1) can be written as

(2) $$\gamma =\frac{K_{I}^{2}}{2E}$$

where γ is the unit surface fracture surface energy (J/mm^2^).

The law of crushing states that the effective energy consumed during crushing is proportional to the newly increased surface area of the particles after crushing. The energy per unit area of the sample, which is the surface energy, can be expressed as

(3) $$\gamma =\frac{U}{\Delta S}$$

where U is the effective energy consumed by crushing (J); Δ*S* is the new surface area after crushing (m^2^). Based on Equations (2) and (3), the relationship between fracture toughness, the newly added surface area and the elastic modulus can be expressed as [29]

(4) $$K_{I}^{2}=c_{1}\frac{E}{\Delta S}$$

where the fitting constant $c_{1}=2U$ signifies that different crushing methods will consume different amounts of effective energy.

According to the standard GB/T 23561.12-2010 [41], the hardness coefficient, denoted as f, represents the hardness of coal, and it is a comprehensive index of its ability to resist external force damage.

(5) $$f=\frac{20n}{l}$$

where f is the hardness coefficient; n is the number of impacts for each sample; *l* is the measured height of sieved coal powder for each group of samples (mm).

The crushing energy exerted by the falling weight on the sample is $U=m_{l}ghn$. The crushing energy per unit mass of the sample is denoted as $A=m_{l}ghn/m_{0}$. The work consumed to produce new unit surface area after crushing, which is the crushing work ratio, can be expressed as

(6) $$W=\frac{U}{m_{0}\Delta S}$$

The crushing work ratio is an important indicator reflecting the rock’s resistance to external force crushing, where W is the crushing work ratio (J/(g·m^2^)); $m_{0}$ represents the mass of the sample; ΔS is the new apparent area after the sample is broken.

The bottom area of the measuring cylinder is considered constant. Measuring the height of the sample under the sieve (l) is equivalent to measuring the total volume of the sample under the sieve (V). In Equation (5), the relationship between the crushing work ratio and the hardness coefficient is

(7) $$f\propto \frac{U}{V}=\frac{\Delta S}{V}m_{0}W$$

where V=m/ρ is the volume of sample under the sieve with a certain particle size; ρ is the sample density.

It is assumed that the initial volume of coal rock with a uniform grain size is $V_{0}$, and the granularity is $2R_{0}$. Therefore, the particle size distribution can be represented as

(8) $$\psi \left(r\right)=\delta \left(r-R_{0}\right)$$

After the sample is crushed, let V be the volume under the sieve, and m be the mass under the sieve. The particle size distribution function is denoted as $\varphi \left(r\right)$, which satisfies $\int_{0}^{R}\varphi \left(r\right)dr=1$. The new area of the sample under the sieve before and after crushing is

(9) $$\Delta S=S_{n}-S_{m}=\int_{0}^{R_{\max}}\varphi \left(r\right)\hat{\Sigma }\left(r\right)Vdr-\hat{\Sigma }\left(R_{0}\right)V$$

where $R_{\max}$ is the maximum particle radius of the sample particles before and after crushing; $S_{m}$, $S_{n}$ are the apparent areas before and after coal crushing, respectively; $\hat{\Sigma }$ is the apparent specific surface area of a single particle $\left(\hat{\Sigma }=\hat{S}/\hat{V}\right)$; $\hat{S}$ is the apparent area of a single particle; $\hat{V}$ is the volume of a single particle. Generally, the maximum particle size after crushing will be smaller than the particle size before crushing. For non-existent particle sizes, the particle size distribution function value is zero.

The apparent specific surface area $\hat{\Sigma }\left(r\right)$ decreases with the increase in sample particle size. The newly added surface area is mainly determined by the smaller particle size. Thus, it can be assumed that the newly added area is approximately equal to the particle size surface area after crushing. Based on Equations (7) and (9), the relationship between the crushing work ratio and the hardness coefficient is

(10) $$f\propto m_{0}W\int_{0}^{R_{\max}}\varphi \left(r\right)\hat{\Sigma }\left(r\right)dr=\frac{c_{1}}{\bar{d}}m_{0}W$$

where $m_{0}W$ is determined by the inherent properties of coal rock; $\bar{d}$ is the mean equivalent diameter of particles smaller than $2R_{\max}$; $c_{1}$ is the shape factor for a sphere $c_{1}=6$. The mean equivalent diameter under the sieve $\bar{d}$ can be expressed as

(11) $$\begin{array}{l} \frac{1}{\bar{d}}=\int_{0}^{R_{\max}}\varphi \left(r\right)\frac{1}{d}dr=\sum \frac{\gamma_{i}}{d_{i}},\\ \gamma_{i}=\frac{m_{i}}{m},m=\sum m_{i},i=1,2,\cdots \end{array}$$

where m is the total mass of particles under the sieve; $m_{i}$ is the mass under the sieve with a particle size of $d_{i}$; $\gamma_{i}$ is the mass proportion of the particle size of $d_{i}$.

It can be observed in Equations (4) and (6) that under the condition of crushing energy U on the particles, the relationship between fracture toughness and crushing work ratio is

(12) $$K_{I}^{2}=2Em_{0}W$$

Based on Equations (10) and (12), it can be seen that the relationship between fracture toughness and the robustness coefficient is

(13) $$K_{I}^{2}\propto f\bar{d}E$$

where $\bar{d}$ is the mean equivalent diameter of the particles under the sieve.

Based on Equations (7), (11) and (13), the fracture toughness can be obtained as

(14) $$K_{I}^{2}=c_{2}f_{KIC},f_{KIC}=\frac{\rho }{m}\bar{d}E=\frac{\rho E}{\sum \frac{m_{i}}{d_{i}}}$$

where $f_{KIC}$ is a parameter linearly related to the square of fracture toughness; $c_{2}$ is a fitting constant. Based on Equation (14), it can be observed that fracture toughness can be quickly determined based on the particle crushing experiment.

According to the standard GB/T 2565-2014 [42], the Hardgrove grindability index (HGI) of coal is directly proportional to the quality under the sieve.

(15) HGI∝m

Based on Equations (14) and (15), the relationship between fracture toughness and HGI can be approximated as

(16) $$K_{I}^{2}\propto \frac{\rho }{HGI}\bar{d}E$$

This result also indicates that fracture toughness has a strong correlation with 1/HGI [5].

## 3. Materials and Methods

### 3.1. Sample Preparation and Equipment

**Figure 1:**
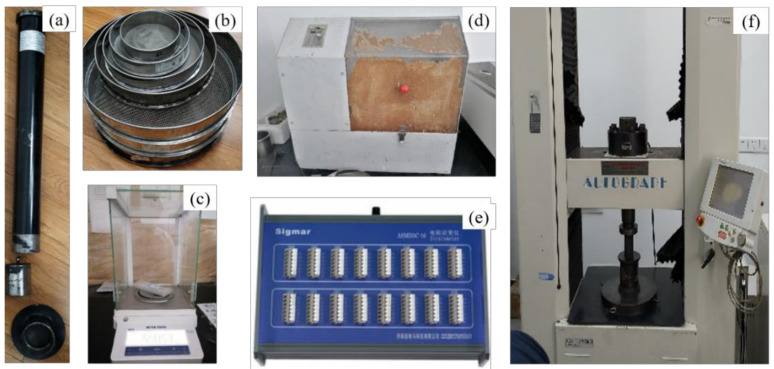
Part of the experimental equipment: (**a**) Coal robustness coefficient measuring instrument; (**b**) Sample dividing sieve; (**c**) Electronic balance; (**d**) Ball mill; (**e**) Strain gauge; (**f**) AG–250 kN-IS testing machine.

Drop weight experiments (DWE), grinding experiments (GCE), uniaxial compressive strength experiments (UCS) and three-point bending experiments (TPB) were conducted using marble, sandstone, non-protruding coal and protruding coal as the test materials. The experimental equipment used is depicted in Figure 1.

The various test types conducted on the four coal rocks were enumerated, and the results are presented in Table 1.

The dimensions of the four coal and rock samples utilized in different experiments are outlined in Table 2.

**Figure 2:**
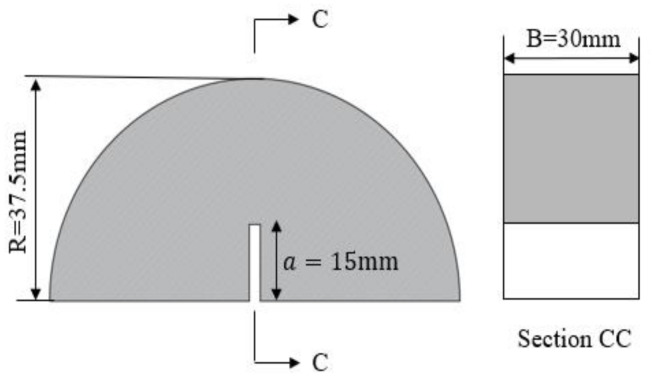
Geometry and loading configuration of the SCB specimen.

The schematic diagram of the NSCB sample size recommended by the ISRM was utilized to measure the Type I static fracture toughness, as depicted in Figure 2.

The sample numbers utilized for different experiments are displayed in Table 3.

### 3.2. Experimental Procedure

- Drop weight experiment

The drop weight experiment procedures refer to “(GB/T 23561.12-2010) [41]”. Raise the weight with a mass of 2.4 kg to a height of 600 mm and drop it freely to impact each sample five times. Then, sieve and measure the mass of different particle size ranges after the coal and rock particles are crushed.

The drop weight experiment procedures follow the guidelines outlined in “(GB/T 23561.12-2010) [41]”. The process involves raising a weight with a mass of 2.4 kg to a height of 600 mm and freely dropping it to impact each sample five times. Subsequently, the coal and rock particles are crushed, sieved, and the mass of different particle size ranges is measured.

- Grinding experiment

The grinding experimental procedures adhere to the “(GB/T 2565-2014) [42]”. The process involves using a ball mill for grinding experiments at various rotation speeds: 150 r/min for 5 min; 250 r/min for 2 min; and again at 250 r/min for another 2 min. Subsequently, the material is sieved, and the mass is measured in different particle size ranges after grinding.

**Figure 3:**
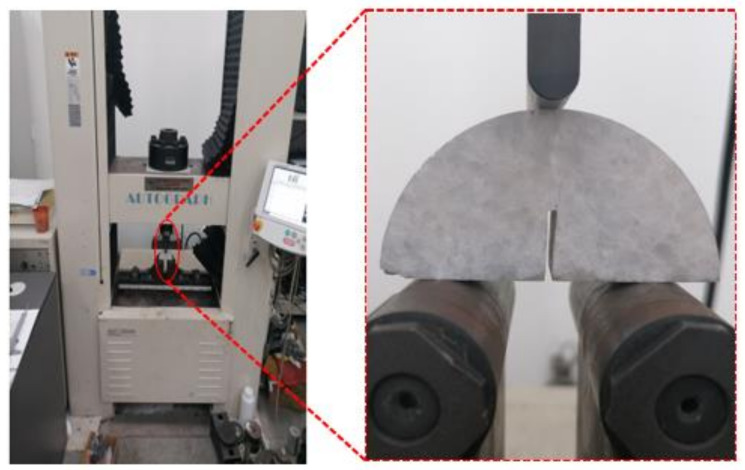
Three-point bending experiment loading method.

- Mechanical properties of coal and rock
- The experimental procedures for measuring the uniaxial compressive strength, elastic modulus and Poisson’s ratio utilize a Φ50 × 100 standard cylindrical specimen. These procedures follow the “(GB/T 23561.7-2009) [43]” and “(GB/T 23561.8-2009) [44]”. Displacement control was employed during loading, with a loading rate set at 0.1 mm/min.
- Three-point bending experiment
- Three-point bending experiments were conducted using NSBN specimens, following the experimental procedures described in the references [8,29]. The loading rate employed was 0.1 mm/min, and the loading method is illustrated in Figure 3.

## 4. Results

### 4.1. Drop Weight and Grinding Experiment

**Figure 4:**
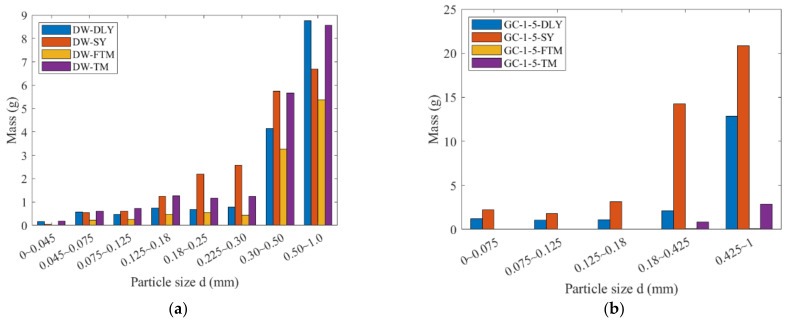

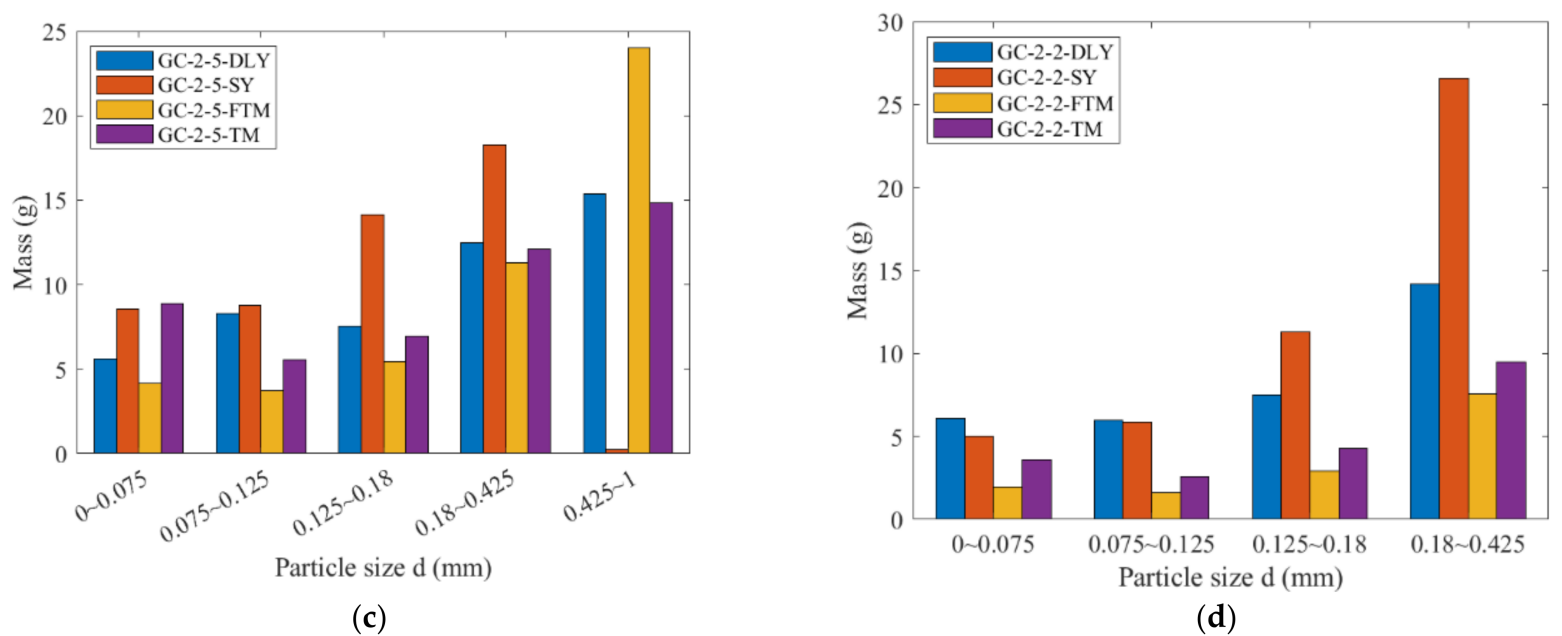
The mass of each particle size under the sieve: (**a**) DW-5; (**b**) GC-1-5; (**c**) GC-2-5; (**d**) GC-2-2.

Four types of coal rocks were utilized to perform the drop weight and grinding experiments. The mass distribution under the sieve after crushing of the samples is depicted in Figure 4.

The mass distribution under the sieve can be analyzed using the Gates–Gaudin–Schuhmann (GGS) model.

(17) $$\Phi ={\left(\frac{d}{d_{\max}}\right)}^{\beta }$$

**Figure 5:**
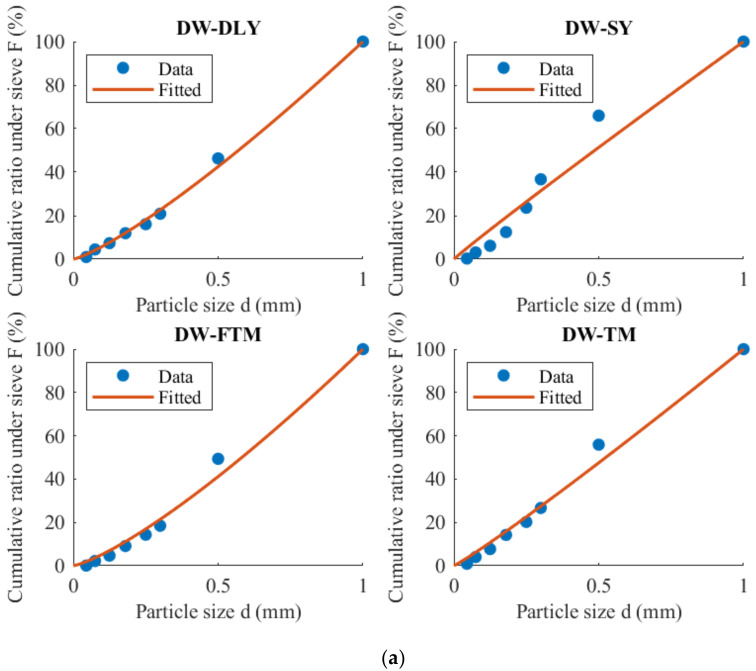

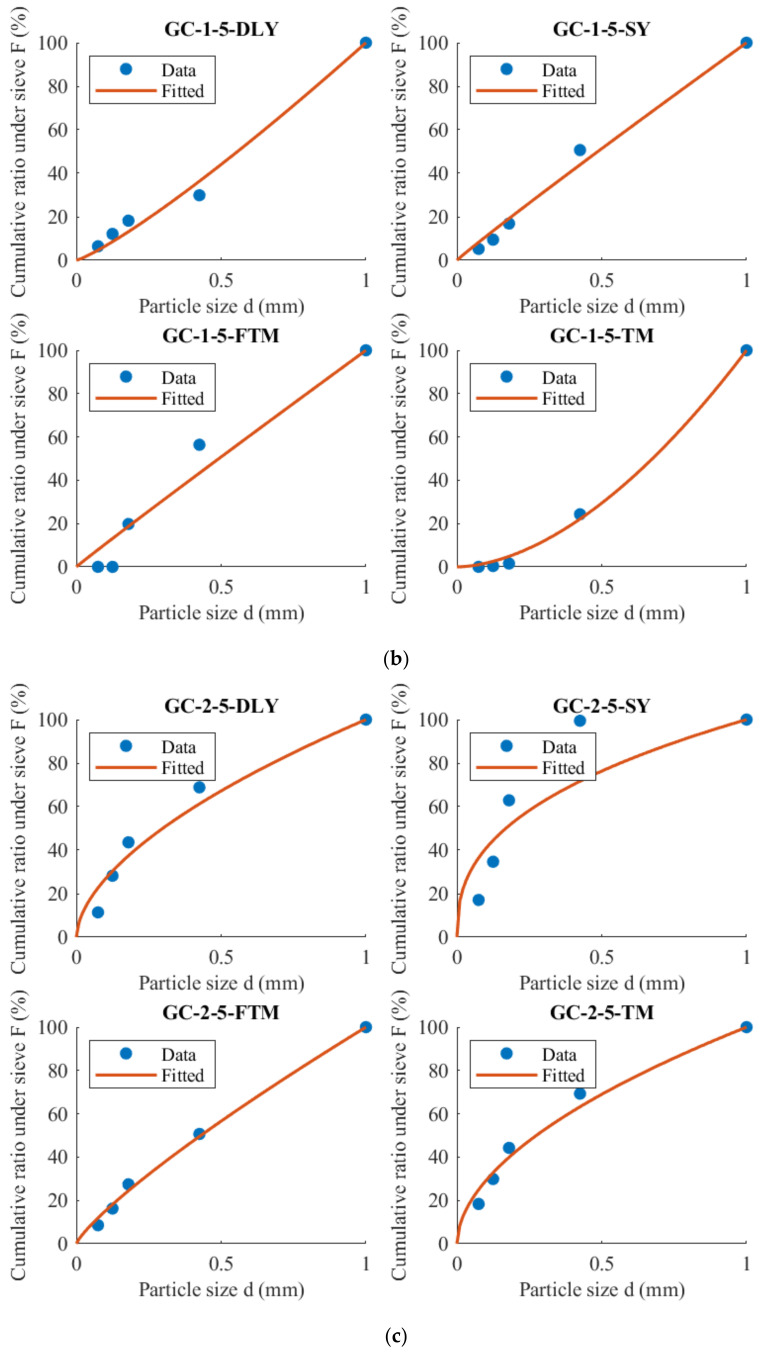

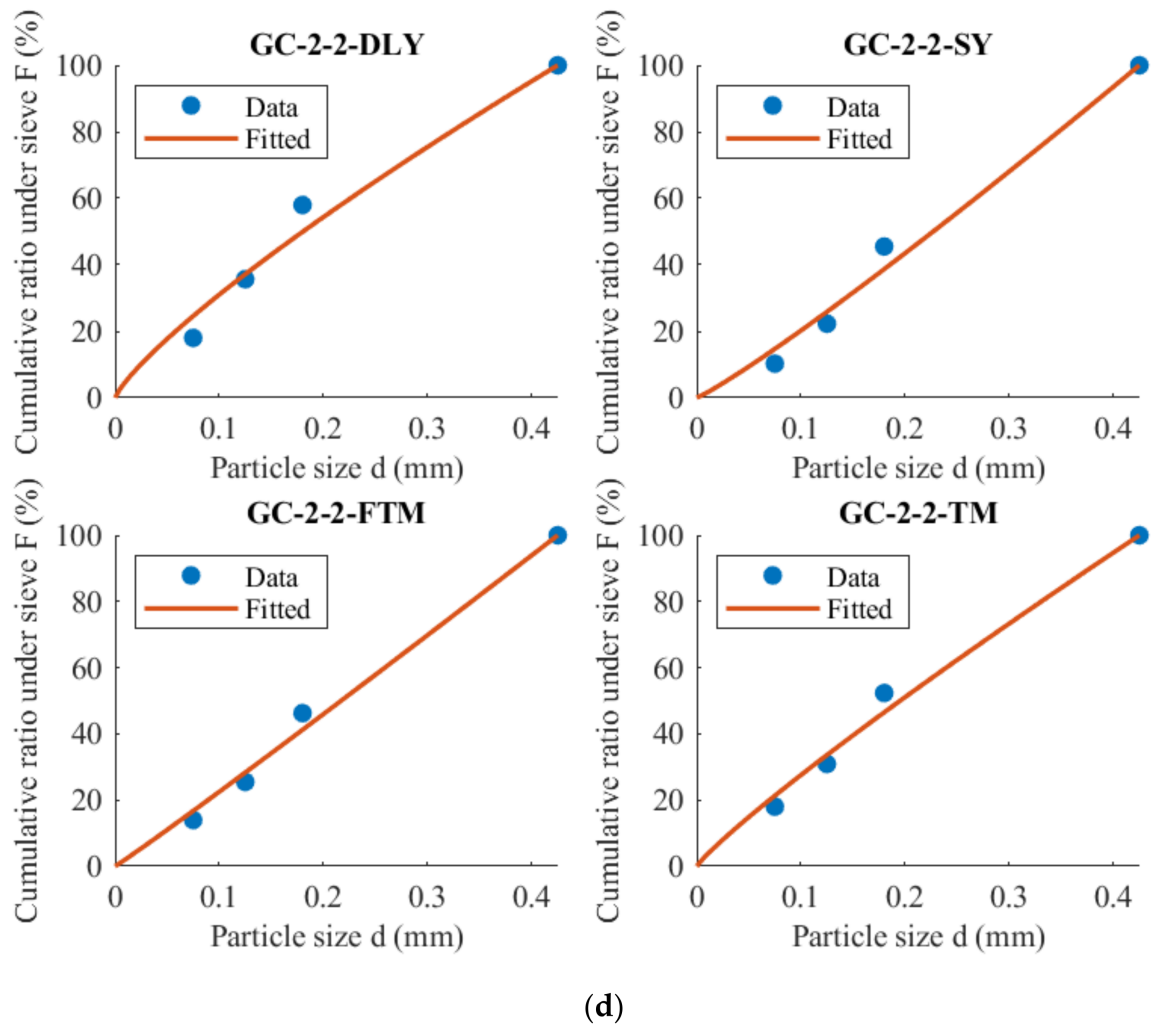
Cumulative mass distribution under sieve: (**a**) DW-5; (**b**) GC-1-5; (**c**) GC-2-5; (**d**) GC-2-2.

Equation (17) is utilized to fit the mass distribution under the sieve under various conditions, and the results are depicted in Figure 5. The fitting outcomes and coefficient of determination (R^2^) are summarized in Table 4.

### 4.2. Uniaxial Compression Experiment

**Figure 6:**
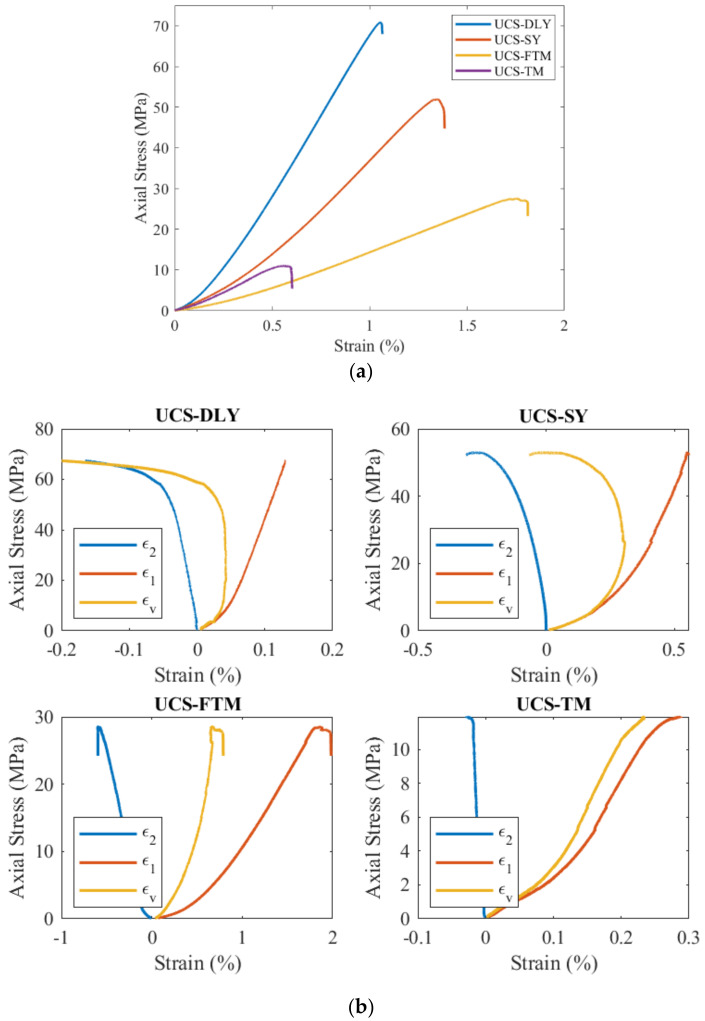
Stress−strain curves of four types of coal rocks: (**a**) Axial strain and stress; (**b**) Strain power.

The uniaxial compressive strength of coal and rock was experimentally determined using the AG–250 kN-IS testing machine. Strain gauges were employed to measure the deformation of coal and rock during uniaxial compression, where $\varepsilon_{1}$ represents longitudinal deformation; $\varepsilon_{2}$ represents transverse deformation; and $\varepsilon_{v}=\varepsilon_{1}+2\varepsilon_{2}$ represents volumetric strain. The stress–strain curve measured is depicted in Figure 6. Based on the figure, the elastic modulus, Poisson’s ratio and peak strength of the four coal rocks can be inferred.

### 4.3. Three-Point Bending Experiment

**Figure 7:**
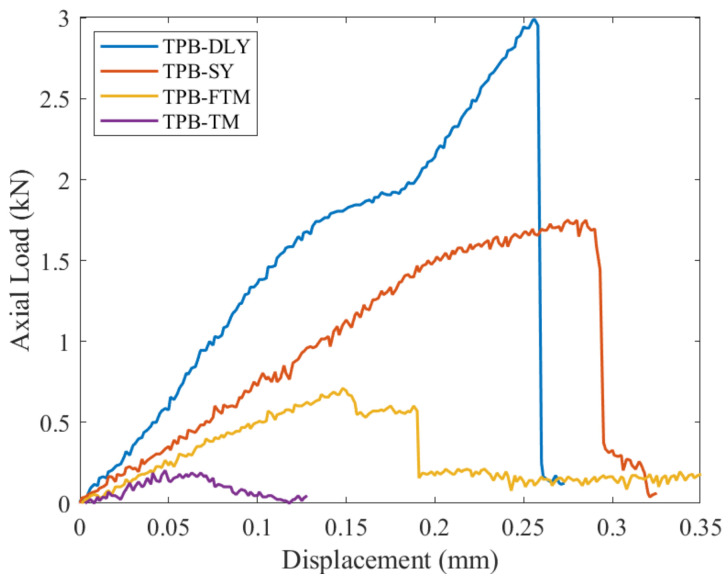
Load–displacement relationship.

Three-point bending fracture experiments were conducted on marble, sandstone, non-outburst raw coal and outburst raw coal. The load–displacement relationship of the NSCB specimen is depicted in Figure 7.

The calculation formula for determining Type I static fracture toughness during experimentation is as follows [8]:

(18) $$K_{I}=\frac{P_{\max}\sqrt{\pi a}}{2RB}Y$$

where $P_{\max}$ is the peak load at specimen failure (kN); a is the artificial prefabricated crack length (mm); R and B are the radius and thickness of the specimen, respectively (mm); Y is a dimensionless stress intensity factor, which is related to the length of artificial prefabricated cracks and the support spacing of the specimen during the experiment, calculated using the formula

(19) $$\begin{array}{c} Y=-1.297+9.516\left[S/\left(2R\right)\right]\\ -\left\{0.47+16.457\left[S/\left(2R\right)\right]\right\}\alpha \\ +\left\{1.071+34.401\left[S/\left(2R\right)\right]\right\}\alpha^{2} \end{array}$$

where α=a/R is the dimensionless prefabricated crack length; S is the distance between the two support points where the specimen is loaded during the experiment; and S/2R is the dimensionless support spacing (where S = 50 mm for this experiment). The uniaxial compressive strength $\sigma_{c}$, elastic modulus E, Poisson ratio ν, three-point bending peak load *P*_max_, fracture toughness of coal and rock obtained in Figure 6 and Figure 7 and Equation (18) are summarized in Table 5.

**Figure 8:**
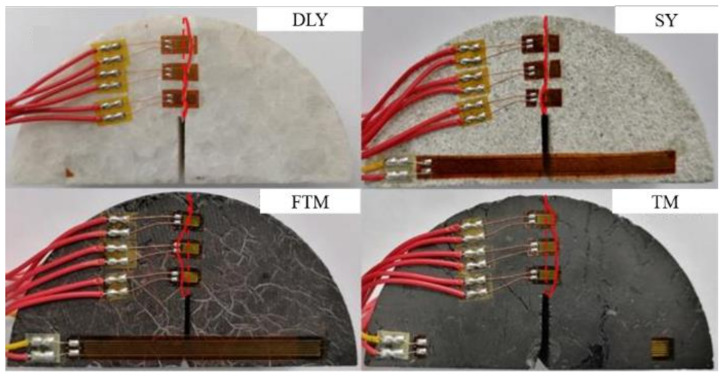
Rock fracture results following the fracture toughness experiment.

The failure and fracture conditions of the four types of specimens are depicted in Figure 8.

Figure 8 reveals that, as the load increases in marble and sandstone, the crack expansion path almost coincides with the straight line where the prefabricated crack is located. However, as the load increases for non-outburst raw coal and outburst raw coal, the crack expansion path deviates from the straight line where the prefabricated crack is located. This deviation is attributed to the greater impact of pore and crack development in coal on the crack expansion path. The actual area of newly opened cracks in coal samples differs from the crack opening areas in marble and sandstone.

## 5. Discussion

### 5.1. Distribution of New Area

After crushing, the newly added surface area of each particle size under the screen comprises two primary components. One part is the mass occupied by each particle size after crushing; the other part is the newly added area of the corresponding particle size unit mass crushed. The new surface area corresponding to each particle size is the product of these two components.

The mass distribution of the particles after crushing represents the mass occupied by each particle size. The law of crushing indicates that finer particles entail greater energy consumption per unit mass of particles. When the energy is minimal, particles may not undergo destruction. Drawing an analogy to the blackbody radiation law, it is hypothesized that there exists a relationship between the particle size after crushing and the new surface area:

(20) $$f_{s}=\frac{c}{d^{3}}\frac{1}{e^{\frac{1}{dE_{0}}}-1}$$

where $f_{s}$ indicates the distribution of the new area; c is the fitting constant; $E_{0}$ is a fitting constant related to the crushing energy. The larger the $E_{0}$, the greater the intensity of the crushing energy and the greater the new surface area of the particles.

**Figure 9:**
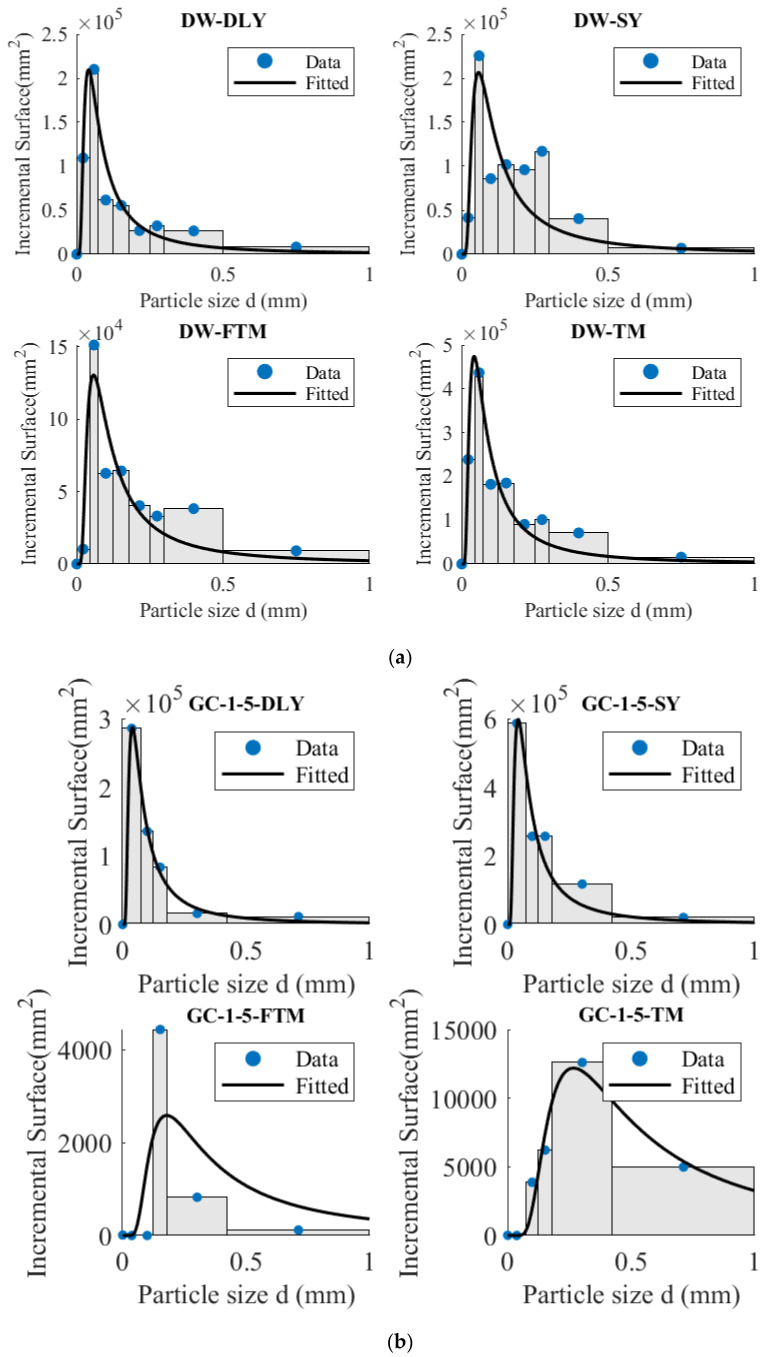

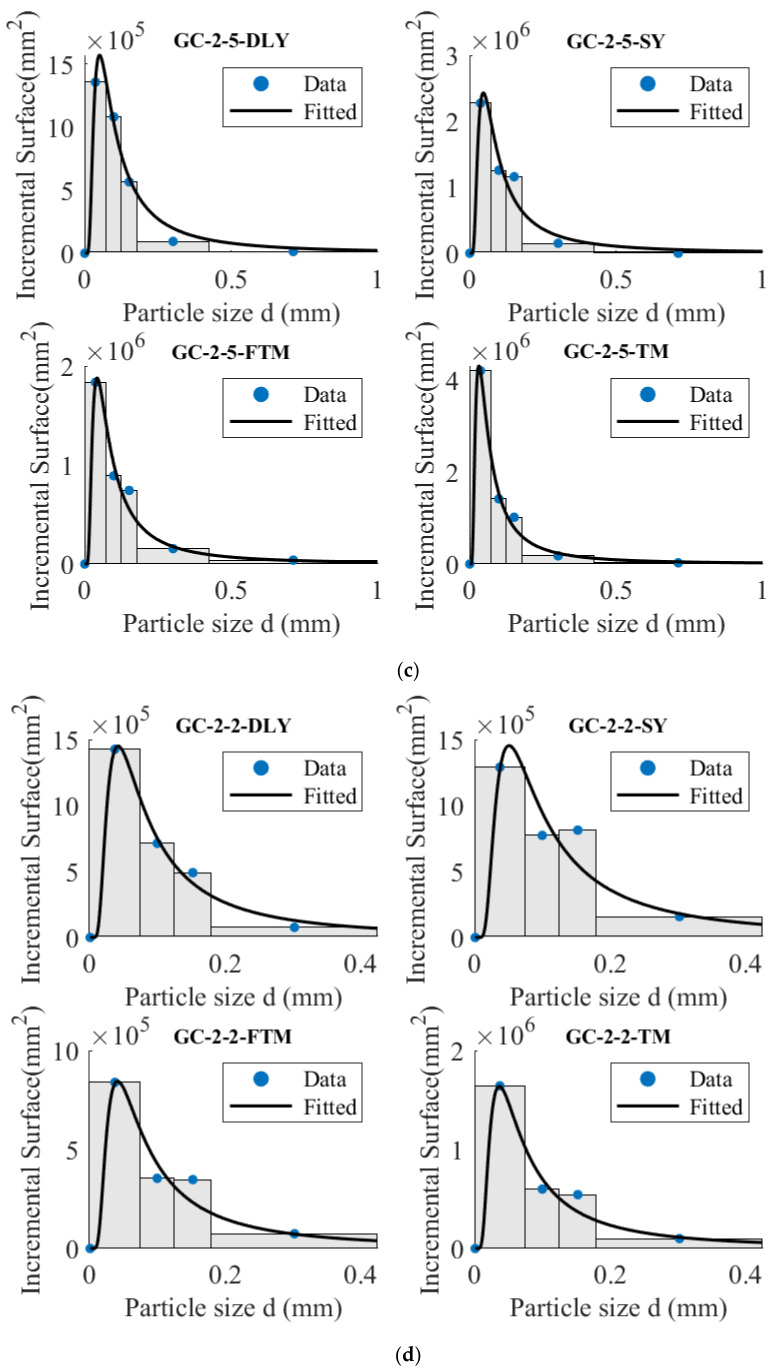
Distribution of new area after hammer drop, grinding and crushing. (**a**) DW-5; (**b**) GC-1-5; (**c**) GC-2-5; (**d**) GC-2-2.

Equation (9) was utilized to calculate the newly added surface area, while Equation (20) was employed to fit the newly added surface area. The distribution of the new area after the coal and rock particles are subjected to hammer drop and grinding is illustrated in Figure 9, with the fitting parameters presented in Table 6. The results demonstrate that Equation (20) adeptly captures the relationship between the newly added surface areas.

Equation (20) establishes the distribution mode of new surface areas after particle breakage, which is influenced by the initial particle size and the energy (*E*_0_) of a single broken particle. It is evident in Table 6 that, in comparison to non-outburst coal, the parameter values (*E*_0_) associated with crushing energy in outburst coal are smaller, suggesting that outburst coal tends to fracture under lower energy conditions. Furthermore, the distribution of crushed coal particles holds significance for coal crushing applications.

### 5.2. Determining Fracture Toughness of Coal

**Figure 10:**
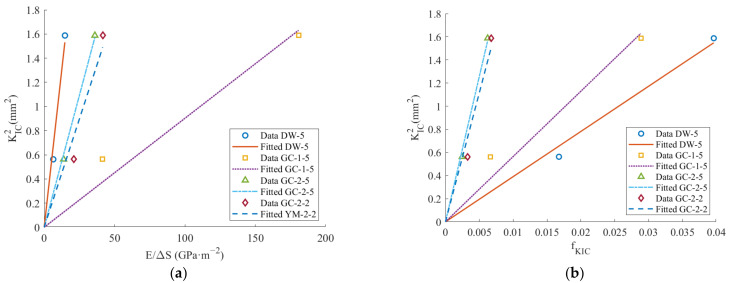
Fracture toughness fitting curve: (**a**) K_IC_-S method; (**b**) K_IC_-f method.

The data for marble and sandstone were selected to fit the relationship between fracture toughness and new surface area according to Equation (4), while the relationship between fracture toughness and $f_{KIC}$ was fitted according to Equation (14). The approach of calculating coal fracture toughness using the fitting results of Equation (4) is denoted as the K_IC_-S method, while the method employing Equation (14) is termed the K_IC_-f method. The fracture toughness fitting curves are illustrated in Figure 10.

Figure 10 illustrates that the ratio of the square of the elastic modulus to the fracture toughness is consistent with a linear relationship with the newly added surface area. Moreover, the square of the fracture toughness is also proportional to $f_{KIC}$. The fitting parameters of Equations (4) and (14) are presented in Table 7.

**Figure 11:**
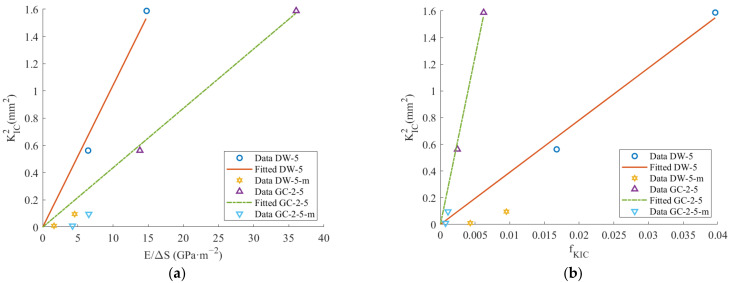
Determining the fracture toughness of coal: (**a**) K_IC_-S method; (**b**) K_IC_-f method.

The fitting results obtained from DW-5 and GC-2-5 were utilized to estimate the fracture toughness of coal, as depicted in Figure 11.

The estimation results of fracture toughness are summarized in Table 8. In Table 8, Ce-V represents the fracture toughness of coal tested in the three-point bending experiment; DW-E represents the fracture toughness of coal determined using drop weight experimental data; and GC-E represents the fracture toughness of coal determined using grinding experimental data.

Figure 11 and Table 8 illustrate that by employing both K_IC_-S and K_IC_-f methods, the results obtained from measuring sandstone and marble samples with undeveloped internal cracks are largely consistent with those measured by the three-point bending test. The maximum error in the test results is 9%, with a minimum of 0.5%. However, when measuring non-outburst raw coal and outburst raw coal, discrepancies are observed between the results obtained from K_IC_-S and K_IC_-f methods compared to those from the three-point bending test. The discrepancy arises because the NSCB coal specimen inherently contains numerous cracks, leading to inconsistencies in the crack expansion direction during the three-point bending test compared to the direction of the prefabricated cracks [29]. The K_IC_-S method and K_IC_-f method are employed to determine the fracture toughness of coal at smaller scales. Hence, a disparity exists between the fracture toughness of coal tested using semi-discs and that tested using particles. As the initial particles are fully ground, most particles have a maximum size smaller than the initial particle size, and the newly added area is significantly larger than the initial area. Consequently, fracture toughness can be determined more accurately. However, when experimenting with the fracture toughness of coal, the developed crack structure of coal leads to the actual new area of coal fracture being smaller than the calculated value. This leads to lower fracture toughness values obtained from the three-point bending experiment compared to those determined using particles. Additionally, due to the varying crack structures of coal across different particle sizes, the estimated fracture toughness results, using materials with different particle sizes, exhibit inconsistencies. Hence, further research on the fracture toughness of coals with different particle sizes is warranted.

## 6. Conclusions

Due to the bedding and fissures developed in coal, the preparation of standard samples becomes challenging, potentially resulting in inaccurate experimental results for Mode-I fracture toughness. Meanwhile, the hardness coefficient is relatively straightforward to measure. Therefore, this paper investigates the relationship between the f value and fracture toughness. Furthermore, a method is proposed to determine Mode-I fracture toughness, with experimental verification of its accuracy. The distribution model of the newly added surface area was proposed based on the observation that finer particles require greater energy consumption for crushing per unit mass. The key conclusions are as follows.

1. The hardness coefficient of coal rock varies directly with the crushing work ratio and inversely with the average equivalent diameter, as determined by crushing energy;
2. The square of the fracture toughness of coal and rock is directly proportional to the crushing work ratio, inversely proportional to the newly added area, directly proportional to the mean equivalent diameter and directly proportional to the hardness coefficient;
3. The Mode-I fracture toughness of coal and rock can be quickly determined through the density, the equivalent diameter after crushing and the elastic modulus;
4. Considering that smaller particle sizes are more resistant to breakage, the paper establishes the distribution mode of new surface areas after particle breakage, which is influenced by the initial particle size and the energy of a single broken particle. This law further elucidates that smaller particles necessitate more energy for crushing.

## Figures and Tables

**Table 1:** Experiment types.

Experiment Type	Condition	Number
DWE	The weight drops five times.	DW-5
GCE	The rotation speed is 150 r/min; the duration is 5 min.	GC-1-5
GCE	The rotation speed is 250 r/min; the duration is 5 min.	GC-2-5
GCE	The rotation speed is 250 r/min; the duration is 2 min.	GC-2-2
UCS	The loading rate is 0.1 mm/min.	UCS
TPB	The loading rate is 0.1 mm/min.	TPB

**Table 2:** Sample dimensions for different experiments.

Sample	Sample Number	DW-5	GC-1-5	GC-2-5	GC-2-2	UCS	TPB
Marble	DLY	Particle size 1–2 mm 50 g/portion	Particle size 1–2 mm 50 g/portion	Particle size 1–2 mm 50 g/portion	Particle size 0.425–1 mm 50 g/portion	Φ50 × 100	NSCB a = 15 m R = 37.5 mm b = 30 mm
Sandstone	SY						
Non-outburst raw coal	FTM						
Outburst raw coal	TM						

**Table 3:** Sample numbers used for experiments.

Sample	DW-5	GC-1-5	GC-2-5	GC-2-2	UCS	TPB
Marble	DW-DLY	GC-1-5-DLY	GC-2-5-DLY	GC-2-2-DLY	UCS-DLY	TPB-DLY
Sandstone	DW-SY	GC-1-5-SY	GC-2-5-SY	GC-2-2-SY	UCS-SY	TPB-SY
Non-outburst raw coal	DW-FTM	GC-1-5-FTM	GC-2-5-FTM	GC-2-2-FTM	UCS-FTM	TPB-FTM
Outburst raw coal	DW-TM	GC-1-5-TM	GC-2-5-TM	GC-2-2-TM	UCS-TM	TPB-TM

**Table 4:** Cumulative particle size distribution under sieve.

Sample	DW-5		GC-1-5		GC-2-5		GC-2-2	
	β	R^2^	β	R^2^	β	R^2^	β	R^2^
Marble	1.233	0.997	1.183	0.988	0.573	0.959	0.809	0.973
Sandstone	0.961	0.965	0.962	0.990	0.389	0.804	1.105	0.984
Non-outburst raw coal	1.280	0.990	0.978	0.954	0.821	0.995	1.031	0.991
Outburst raw coal	1.073	0.990	1.764	0.997	0.535	0.978	0.891	0.987

**Table 5:** Basic mechanical parameters of coal and rock.

Sample	*ρ* g/cm³	*σ_c_*/MPa	*E*/GPa	*ν*	*P*_max_/kN	*K*_ΙC_/MPa·m^1/2^
Marble	2.74	70.82	7.84	0.20	3.00	1.26
Sandstone	2.49	51.88	4.62	0.17	1.76	0.75
Non-outburst raw coal	1.50	27.50	1.86	0.27	0.75	0.31
Outburst raw coal	1.40	10.93	2.12	0.10	0.24	0.10

**Table 6:** New surface area distribution fitting results.

Sample	DW-5			GC-1-5			GC-2-5			GC-2-2		
	*c*	*E*_0_	R^2^	*c*	*E*_0_	R^2^	*c*	*E*_0_	R^2^	*c*	*E*_0_	R^2^
Marble	232	0.116	0.904	294	0.113	0.997	3362	0.145	0.994	1725	0.119	0.991
Sandstone	639	0.163	0.711	780	0.122	0.948	4062	0.133	0.959	3048	0.143	0.911
Non-outburst raw coal	406	0.164	0.846	234	0.505	0.548	2416	0.122	0.980	950	0.116	0.958
Outburst raw coal	597	0.121	0.934	3672	0.753	0.950	2288	0.091	0.994	1277	0.103	0.976

**Table 7:** The fracture toughness parameters determined.

Experiment	DW-5	GC-1-5	GC-2-5	GC-2-2
	c_1_	R^2^	c_2_	R^2^
DW-5	0.104	0.999	39	0.999
GC-1-5	0.009	0.999	56	0.999
GC-2-5	0.043	0.999	251	0.999
GC-2-2	0.035	0.999	223	0.999

**Table 8:** Estimation results of fracture toughness.

**Sample**	**Ce-V**	**K_IC_-S**		**K_IC_-f**	
		**DW-E**	**GC-E**	**DW-E**	**GC-E**
DLY	1.26	1.241	1.254	1.244	1.252
SY	0.75	0.820	0.776	0.809	0.785
FTM	0.31	0.688	0.535	0.610	0.524
TM	0.10	0.409	0.430	0.410	0.432

## Data Availability

Data are contained within the article.
